# Regulation of Organic Anion Transporting Polypeptides (OATP) 1B1- and OATP1B3-Mediated Transport: An Updated Review in the Context of OATP-Mediated Drug-Drug Interactions

**DOI:** 10.3390/ijms19030855

**Published:** 2018-03-14

**Authors:** Khondoker Alam, Alexandra Crowe, Xueying Wang, Pengyue Zhang, Kai Ding, Lang Li, Wei Yue

**Affiliations:** 1 Department of Pharmaceutical Sciences, College of Pharmacy, University of Oklahoma Health Sciences Center, Oklahoma City, OK 73117, USA; khondoker-Alam@ouhsc.edu (K.A.); Alexandra-Crowe@ouhsc.edu (A.C.); 2 Center for Computational Biology and Bioinformatics, Indiana Institute of Personalized Medicine, Department of Medical and Molecular Genetics, Indiana University School of Medicine, Indianapolis, IN 46202, USA; wangxueyinghrbeu@foxmail.com (X.W.); zhangpe@imail.iu.edu (P.Z.); Lang.Li@osumc.edu (L.L.); 3 Department of Biostatistics and Epidemiology, College of Public Health, University of Oklahoma Health Sciences Center, Oklahoma City, OK 73126, USA; kai-ding@ouhsc.edu; 4 Department of Biomedical Informatics, Ohio State University, Columbus, OH 43210, USA

**Keywords:** OATP1B1, OATP1B3, transcription, post-translation, protein degradation, lysosome inhibitor, proteasome inhibitor

## Abstract

Organic anion transporting polypeptides (OATP) 1B1 and OATP1B3 are important hepatic transporters that mediate the uptake of many clinically important drugs, including statins from the blood into the liver. Reduced transport function of OATP1B1 and OATP1B3 can lead to clinically relevant drug-drug interactions (DDIs). Considering the importance of OATP1B1 and OATP1B3 in hepatic drug disposition, substantial efforts have been given on evaluating OATP1B1/1B3-mediated DDIs in order to avoid unwanted adverse effects of drugs that are OATP substrates due to their altered pharmacokinetics. Growing evidences suggest that the transport function of OATP1B1 and OATP1B3 can be regulated at various levels such as genetic variation, transcriptional and post-translational regulation. The present review summarizes the up to date information on the regulation of OATP1B1 and OATP1B3 transport function at different levels with a focus on potential impact on OATP-mediated DDIs.

## 1. Introduction

Membrane transporter proteins play important roles in facilitating the translocation of endogenous compounds and xenobiotics across biological membranes. The organic anion transporting polypeptides (OATPs) are a family of transporters and a subgroup of the solute carrier organic anion (SLCO) transporter superfamily [1]. Both *OATP1B1* and *OATP1B3* genes are located on the short arm of chromosome 12 (gene locus 12p12) [2]. OATP1B1 and OATP1B3 proteins share similar amino acid sequences with 80% homology [3]. OATP1B1 and OATP1B3 are both highly expressed in normal human liver and localized on the basolateral membrane of hepatocytes [3,4]. However, OATP1B1 and OATP1B3 have different zonal expression pattern in the liver. OATP1B3 is expressed primarily around the central vein of hepatic lobules [3], while OATP1B1 has a diffuse expression pattern throughout the liver sections [3,5]. 

OATP1B1 and OATP1B3 mediate the hepatic uptake of many clinically important drugs (e.g., the 3-hydroxy-3-methylglutaryl-coenzyme (HMG-CoA) reductase inhibitors, anti-diabetics, anti-cancers) and endogenous compounds (e.g., bile acids) [6]. Impaired transport function of OATP1B1 and OATP1B3 due to genetic variation or drug-drug interactions (DDIs) often leads to severe adverse events such as statin-induced rhabdomyolysis. A recent review article has emphasized the importance of OATP1B1 and OATP1B3 on statin drug interactions [7]. Because OATP1B1 and OATP1B3 play important roles in transporter-mediated DDIs [8], assessing OATP-mediated DDI potential of new molecular entities has been recommended by US Food and Drug Administration (FDA) and other regulatory agencies [9,10,11]. Several OATP1B1- and OATP1B3-focused review articles have been published, focusing on methodologies of prediction of OATP-mediated DDIs.

## 2. Scope

Since the last decade, a significant amount of knowledge has been gained regarding how competitive OATP inhibition and genetic variation contribute to altered disposition of OATP substrates. Studies especially published recently, demonstrate that transporter function of OATP1B1 and OATP1B3 can also be altered at other levels such as transcriptional or post-translational regulation, or by drugs that affect protein degradation. It is intriguing that therapeutic drugs/new molecular entities that can alter OATP1B1 and OATP1B3 at various levels may have the potential to cause OATP-mediated DDIs. Currently, a systematic review of up-to-date findings on modulation of OATP1B1- and OATP1B3-mediated transport at various levels, particularly in the context of OATP-mediated DDIs, is lacking. Such information would be beneficial for researchers in drug development and regulatory agencies. This article focuses primarily on current progress and knowledge gaps in the function and regulation of OATP1B1- and OATP1B3-mediated transport and implications of such regulation in OATP-mediated DDIs. 

## 3. Substrate Transport Specificity and Transport Mechanism of OATP1B1 and OATP1B3

OATP1B1 and OATP1B3 share common substrates, such as statins [12,13], rifampicin [14], bromosulphophthalein (BSP) [15], bosentan [16], valsartan [17] and olmesartan [18] and endogenous compounds, including bile acids, thyroid hormones, steroid sulfates, glucuronide conjugates and peptides [3,4,19,20]. Some substrates, such as estrone-3-sulfate [12], are transported preferentially by OATP1B1, while others, such as telmisartan [21], peptide deltorphin II [22], the hepatotoxic cyclic peptide amanitin [23], the cardiac glycoside ouabain [24] and cholecystokinin octa-peptide (CCK-8) [25], are transported preferentially by OATP1B3. 

The OATP1B1 and OATP1B3 transport proteins consist of 691 and 702 amino acids, respectively; OATP1B3 has 80% amino acid homology with OATP1B1 [3,4]. OATP1B1 and OATP1B3 have 12 putative transmembrane domains with both termini located within the cytoplasmic side [26,27,28]. It has been reported that specific amino acids in transmembrane domains (TM) 2, 6, 8, 9 and 10 and extracellular loop (ECL) 6 are critical for the transport function of OATP1B1 and OATP1B3 substrates [29,30,31,32]. Replacement of A45 in TM1, L545 in TM10 and T615 in ECL 6 of OATP1B1 with the respective amino acids in OATP1B3 enabled OATP1B1 to transport CCK-8, which is a specific substrate of OATP1B3 [29]. 

Estradiol-17β-glucuronide and estrone-3-sulfate are two substrates that are commonly used for in vitro OATP1B1 transporter function assays [12]. Substituting the TM8 of OATP1B1 with that of OATP1B3 produces a protein that has 18-fold lower affinity for estrone-3-sulfate than does wild-type OATP1B1 and completely abolishes the transportability of estradiol-17β-glucuronide [32]. Replacing the TM9 of OATP1B1 with that of OATP1B3 decreases the affinity for estrone-3-sulfate by about 7.4-fold but does not change the transport kinetics for estradiol-17β-glucuronide [32].

To date, the transport mechanism of OATPs remains unclear. Bicarbonate was first identified as the counter-ion in the transport of taurocholate in rat Oatp expressed in HeLa cells [33]. Another study reported that OATP-mediated transport of its substrate is coupled with bicarbonate efflux [34]. Reduced glutathione (GSH) has been described as the driving force of rodent Oatp1-mediated transport [35]. One study demonstrated that uptake of bile acids by human OATP1B3 is co-transported by glutathione [36]. However, such findings could not be replicated [37].

OATP1B1 and OATP1B3 have been reported as electrogenic transporters whose activity may be strongly affected under circumstances of displacement of local pH [38]. Extracellular pH appears to affect OATP1B1- and OATP1B3-mediated transport. Martinez-Becerra et al., 2011 [38] showed that an extracellular pH of 6.5 stimulates OATP1B3-mediated transport of taurocholate, estradiol-17β-glucuronide and estrone-3-sulfate, compared with a physiological pH of 7.4. Lower extracellular pH also stimulated OATP1B1-mediated transport of estrone-3-sulfate but not taurocholate and estradiol-17β-glucuronide [38]. Further, Leuthold et al. reported that a lower extracellular pH of 6.5 stimulated transport of taurocholate, estrone-3-sulfate, thyroxine and prostaglandin E2 by OATP1B1 and/or OATP1B3, compared with a pH of 8 [34]. 

## 4. Altered Hepatic Disposition of OATP1B1/1B3 Substrates Due to Genetic Variation and Drug-Drug Interactions

Several single nucleotide polymorphisms (SNP) of OATP1B1 and OATP1B3 have been identified (reviewed by Nakanishi et al., 2012) [6]. Altered drug and xenobiotic disposition is often associated with genetic variation of OATP1B1 and OATP1B3. A SNP of SLCO1B1 (OATP1B1-encoding gene) gene (c.521T>C, p.Val174Ala) has significantly reduced transport activity in vitro [39]. In vivo, the SLCO1B1 c.521T>C polymorphism is associated with increased risk of simvastatin-induced myopathy [40]. The SNP c.521T>C (p.Val174Ala) is more common in European-Americans (allelic frequency 14% [39]) and Asian (allelic frequency 10–15% [41]) populations. This genetic polymorphism was reported to increase the plasma exposure of atorvastatin and rosuvastatin in 32 healthy white subjects [42]. Increase in plasma exposure of other OATP1B1 substrates such as fexofenadine, irinotecan, lopinavir, nateglinide, pravastatin and repaglinide were also observed in patients with the homozygous c.521T>C genotype [43,44,45,46,47,48,49].

The molecular mechanism for such decreased transport function of OATP1B1 c.521T>C was believed to be associated with decreased levels of the transport protein on the plasma membrane, based on an in vitro study in HeLa cells showing that the OATP1B1 c.521T>C variant has reduced plasma membrane localization [39]. Two frequent coding, nonsynonymous SNPs of OATP1B3 (T334G, G699A) that are in complete linkage disequilibrium [50] have been reported. In vitro, this OATP1B3 variant protein (i.e., 334G–699A haplotype) has reduced transport activity toward mycophenolic acid glucuronide (MPAG) compared with the reference OATP1B3 protein. The variant has a similar Michaelis-Menten constant (*K*_m_) but a decreased maximal transport velocity (V_max_) [51]. In vivo, the carriers of the 334G allele are associated with a significant increase in exposure to mycophenolic acid (MPA), which is likely secondary to decreased MPAG hepatic uptake and the subsequent reduction in MPA reabsorption through enterohepatic cycling [51]. 

Complete and simultaneous genetic deficiencies of OATP1B1 and OATP1B3 have been reported to be linked to Rotor syndrome, a rare and benign hereditary hyperbilirubinemia [52]. RS is also associated with significantly reduced hepatic uptake of many diagnostic compounds that are OATP substrates, such as ^99m^Technetium-mebrofenin [53]. Indocyanine green (ICG) is a substrate of OATP1B3 [53]. The ICG retention test is widely used for preoperative evaluation of liver function. A recent report indicates that a deficiency in OATP1B3 due to genetic variation is associated with marked delay of ICG clearance [53].

Drugs that are OATP1B1/1B3 inhibitors (e.g., gemfibrozil, cyclosporine A, rifampicin and ritonavir) may cause clinically significant adverse effects, such as myopathy, when co-administered with lipid-lowering statins, which are substrates of OATPs [54,55,56,57]. Such drug-drug interactions (DDIs) may lead to life-threatening rhabdomyolysis in severe cases [58]. In addition, co-administration of OATP inhibitors has been reported to increase the plasma exposure of statins. Co-administration of gemfibrozil and rosuvastatin, a metabolically stable statin [59], resulted in about 1.88- and 2.21-fold increases in the area under the curve (AUC) and peak plasma concentration (C_max_) of rosuvastatin, respectively, in healthy volunteers, presumably by inhibiting the transport function of OATP1B1/1B3 [60]. Immunosuppressant cyclosporine (Cs) A, an inhibitor of OATP1B1 and OATP1B3 [61], has been reported to increase the AUC and C_max_ of rosuvastatin by 7.1- and 10.6-fold, respectively, in patients who underwent heart transplantation [62]. A previous publication thoroughly reviewed potential OATP-mediated DDIs of perpetrator drugs that are inhibitors of OATP1B1 and/or OATP1B3 against 12 drugs that are substrates of OATP1B1/OATP1B3 [63]. Using a similar approach as this previous review, the current review used the PubMed database and key words “drug name and pharmacokinetics” and updated the potential OATP-mediated DDIs of these 12 drugs in the literature from 2008 to 2018. An AUC ratio (AUCR) (with vs. without perpetrator drugs) of greater than 1.25 was used as a cut-off value for in vivo DDI. Only DDI reports not listed in previous review [63] are summarized in Table 1. 

## 5. Altered Expression of OATP1B1 and 1B3 in Pathological Conditions

Though OATP1B1 and OATP1B3 are predominantly expressed in normal human liver, expression of OATP1B1 and OATP1B3 mRNA and immunoreactivity to OATP1B1/1B3 proteins were also detected in various cancers. OATP1B1 was reported to be expressed in tumors of lung, prostate, colon and pancreas [103]. For OATP1B1 expression in hepatocellular carcinoma, some studies reported increased expression while one reported unchanged compared to control [104,105,106]. Recently, a cancer-type OATP1B3 mRNA was identified in cancer cell lines and tissues from lung, colon and pancreatic origin [107,108,109]. Compared to wild-type OATP1B3 that is highly expressed in the liver, the cancer-type OATP1B3 lacks an N-terminus encoding region. The putative protein expression of the cancer-type OATP1B3 is primarily expressed in the cytosol and has minimal transport function when expressed exogenously in cancer cell lines [109,110].

## 6. Transcriptional Regulation of OATP1B1 and OATP1B3

The ontogeny of OATP1B1 and 1B3 expression and mRNA levels has been recently investigated. In a study by Thomson et al., 2016, OATP1B1 and 1B3 expression levels differed at different ages, where OATP1B3 expression fluctuated from high expression at birth to lower levels during the toddler ages and the back up at the pre-adolescent years [111]. OATP1B1 expression however, seemed to be reduced at the younger ages overall [111]. Another report in 2014 found that mRNA expression of OATP1B1 and 1B3 in the liver was significantly reduced at the younger ages when compared to adult expression, where OATP1B1 and 1B3 mRNA expression levels were reduced by 500- and 600-fold in neonates and 90- and 100-fold in infants when compared to adults, respectively [112]. These findings are key in understanding the progression of expression of OATP1B1 and 1B3 as we age and could lead to the higher variability of a drug’s pharmacokinetics and disposition found in the pediatric population. 

Different transcription factors have been reported to regulate the expression of OATP1B1 and OATP1B3. The OATP1B1 promoter is transactivated by hepatic nuclear factor (HNF)1α [113] and 4α [114], liver X receptor (LXR) α [115] and farneosid X receptor (FXR) [115] whereas the OATP1B3 promoter is transactivated by FXR [116], HNF1α [113] and growth hormone- and prolactin-activated transcription factor (STAT5) [116]. OATP1B3 transcription can also be repressed by hepatic nuclear factor (HNF 3β) [105]. The constitutive androstane receptor (CAR) activator phenobarbital decreased the expression of OATP1B3 in human liver slices [117], while the retinoic acid ligand was able to reduce mRNA expression of OATP1B1 in human hepatocytes [118]. Although few are known for OATP1B1 and 1B3, epigenetic mechanisms can also regulate their expression. For example, 5-aza-2′-deoxycytidine, which inhibits DNA methylation, was able to increase the mRNA levels of OATP1B3 in various cancer cell lines [119,120]. Regulation at the transcriptional and epigenetic levels can play a significant role in the total mRNA expression of OATP1B1 and 1B3.

## 7. Post-Translational Regulation of OATP1B1 and OATP1B3

### 7.1. Glycosylation

At the post-translational level, both OATP1B1 and OATP1B3 have been reported to be glycosylated proteins [3,121]. N-linked glycosylation at asparagine (Asn) residues are important for the regulation of membrane transporters such as OATP1B1 and 1B3 [122]. Briefly, oligosaccharides are added to the asparagine residues by oligosaccharyl-transferase enzymes [122]. Three of the terminal glucose residues and at least one mannose residue is removed from the protein in order to prepare the membrane protein for trafficking towards the plasma membrane [123,124]. Glycosylation of OATP1B1 is reported to occur at the second and fifth extracellular loops, while the un-glycosylated portion of the protein is retained in the cytoplasm. Asparagine (Asn) 134 and Asn 516 were reported to be involved in the glycosylation process under basal conditions; however, mutation of Asn 134 also led to additional glycosylation at Asn 503. Simultaneous substitution of these three asparagine residues (Asn 134, Asn 503 and Asn 516) with glutamines resulted in a significant reduction of OATP1B1 transport activity and protein expression on the plasma membrane, indicating that these sites may be important for the expression and/or function of OATP1B1 [121].

The functional consequence of glycosylation on OATP1B3 transport function still remains unclear, as there are few reports of glycosylation sites in OATP1B3. In non-alcoholic steatohepatitis (NASH), a significant loss of glycosylation of OATP1B1 and OATP1B3 was recently reported, suggesting that the loss of glycosylation of OATP1B1 and OATP1B3 may contribute to altered drug disposition in NASH [125]. In a study looking at OATP1B3 expression in HEK293 cells, the authors found two bands on their immunoblot for OATP1B3 and deemed them as highly glycosylated and core-glycosylated forms of OATP1B3 [111]. Confirming this study, it was also found in HeLa cells transfected with OATP1B3 that the glycosylated form of OATP1B3 was found at ~100 kDa and the un-glycosylated form of 1B3 was found at ~70 kDa (similar to molecular weight of OATP1B3) [126]. In the same 2016 Thomson study, it was also reported that levels of the glycosylation also change with age and that glycosylation may play a role in the development of OATP1B3 function as we age [111].

### 7.2. Phosphorylation

Phosphorylation of transporters such as the organic anion transporter (OAT) 1 [127] and 3 [128], OATP2B1 [129] and as well as the efflux transporters MRP2 [130] and P-gp [131] have been shown to be a key regulator of their transport function [122]. Both OATP1B1 and 1B3 have been predicted to be phosphorylated using phosphoproteomic analysis of human liver tissue [132]. Guil et al., 2014 characterized OATP1B3 as a phosphorylated protein in human sandwich-cultured hepatocytes (SCH) [133] and reported that increased phosphorylation of OATP1B3 was associated with the rapid downregulation of OATP1B3 transport function following protein kinase C (PKC) activation with phorbol 12-myristate 13-acetate (PMA) [133]. 

Computational analysis showed that OATP1B1 protein has many putative phosphorylation sites [134]. Although phosphorylation of OATP1B1 has not been characterized up to this date, Hong et al., 2015 demonstrated that PKC activation by PMA also downregulated the transport function of OATP1B1 and was associated with decreased surface expression of OATP1B1 on the membrane [134]. Future studies such as site directed mutagenesis are still warranted to better understand how phosphorylation regulates OATP1B1 and 1B3 transport function and as well as determine how functional significance of modulation of phosphorylation status correlates with the downregulation of transport function. 

### 7.3. Ubiquitination

Ubiquitin is a small, 76-amino-acid protein where it forms an isopeptide bond between a lysine residue on the protein and the carboxyl terminus of ubiquitin [135,136]. Ubiquitination is an important post-translational modification that regulates many cellular processes including signal transduction, cell cycle control and transcriptional regulation through mediating proteasome degradation of proteins and the maintenance of protein homeostasis [137]. A recent study reported that both OATP1B1 and OATP1B3 can be ubiquitin-conjugated in HEK293 cells over-expressing OATP1B1 or OATP1B3 [138]. However, the effects of ubiquitination on OATP1B1 and OATP1B3 transport function have not been demonstrated and further studies are warranted. 

## 8. Regulation of OATP1B1/1B3 Transport Function by Drugs Perturbing Protein Degradation

Protein degradation is a fundamental cell process that regulates the abundance of protein and meets the functional needs of the cell [139]. The ubiquitin-proteasome system (UPS) and lysosomal pathways are the two major mechanisms by which cellular proteins are degraded [140]. The ubiquitin-proteasome system is a major pathway for proteolysis of intracellular proteins (~90%), as well as many membrane proteins [141,142,143,144,145]. The lysosome, a membrane-enclosed organelle inside the cells, contains about 50 different degradative enzymes and is responsible for breaking down of different kinds of biological polymers, including proteins [146]. Lysosomal enzymes are active in an acidic environment (pH ~ 5) [146]. Endocytosis is the major mechanism through which the digestion materials are taken up into the lysosome. 

### 8.1. Regulation of OATP1B1 and OATP1B3 Transport Function by Lysosome Inhibition

Chloroquine, a class 4-aminoquinoline drug used for the treatment of malaria [147], is a widely used lysosome inhibitor in the laboratory to study lysosomal degradation of proteins [148,149]. Beyond its therapeutic use in malaria, clinical applications of chloroquine in the treatment of autoimmune diseases, such as systemic lupus erythematosus and rheumatoid arthritis, have been reported over the past 70 years [150,151,152,153,154]. The use of chloroquine in cancer therapy has drawn great attention. Several clinical trials have evaluated the anticancer properties of chloroquine, either alone [155,156,157] or in combination with other chemotherapy drugs [158,159]. The diphosphate salt of chloroquine is a diprotic weak base (pKa1 = 8.1, pKa2 = 10.2) [160] and can exist in both protonated and un-protonated forms [160]. The un-protonated form of chloroquine can freely diffuse through the biological membrane of organelles. Chloroquine is protonated inside the organelles that are acidic in nature, such as the lysosome [160]. The protonated form of chloroquine cannot diffuse back through the membrane and thus is trapped in acidic organelles [160]. Accumulation of chloroquine inside the lysosome impairs the proteolytic process of the lysosome by increasing the pH of the lysosomal fluid [160]. 

In OATP1B1- and OATP1B3-expressing stable cell lines and human SCH, treatment with lysosome inhibitor chloroquine markedly increased protein levels of OATP1B1 and 1B3, suggesting that the lysosome plays an important role in degradation of OATP1B1 and OATP1B3 [138,161]. The estimated maximum unbound concentration of chloroquine at the inlet to the liver is ~56.8 μM after a 600 mg single dose in human [161]. At concentrations up to 100 µM, acute incubation with chloroquine does not competitively inhibit OATP1B1-mediated substrate transport [161,162]. However, acute incubation with chloroquine at 10 μM inhibited OATP1B3-mediated transport to ~20% of control [162]. 

Pretreatment with chloroquine significantly decreases OATP1B1-mediated transport in transporter-expressing HEK293 stable cell lines and pitavastatin uptake in human SCH [161]. The pretreatment effect of chloroquine on OATP1B3 has not been reported. Monensin and bafilomycin A1, which are reported to inhibit lysosome activity, also inhibit OATP1B1 transport function after pretreatment [161]. 

A pharmacoepidemiological study was conducted to assess the risk of concurrent administration of chloroquine on statin-induced myopathy risk. Concurrent administration of chloroquine significantly increased the statin-related myopathy risk in women compared with the administration of statins (pitavastatin, rosuvastatin, or pravastatin) alone (9.6% vs. 21.9%, relative risk ratio (RR) = 2.28; *p*-value = 0.03) [161]. 

**Figure 1:**
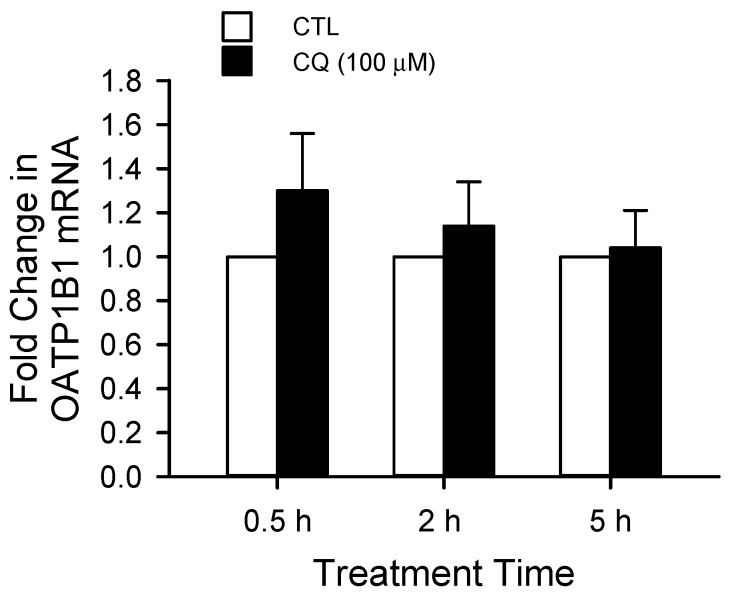
Chloroquine treatment did not affect OATP1B1 (organic anion transporting polypeptides 1B1) mRNA levels in HEK293-OATP1B1 cells. Model-estimated fold change and associated SE in OATP1B1 mRNA levels vs. control are shown at each treatment time. HEK293-OATP1B1 cells were treated with 100 μM chloroquine (CQ) or vehicle control (CTL) for 5 h. OATP1B1 mRNA levels were determined by TaqMan^®^ real-time RT-PCR. OATP1B1 expression relative to that of control was analyzed with the 2^−ΔΔ*C*t^ method [163] using GAPDH as an internal control. The TaqMan^®^ probe and primer sequences (5′–3′) used for human OATP1B1 were TCCTACATGACCCACGTGTGCCACA (probe), CATGTATGAAGTGGTCCACCA (forward primer) and CAAGTAGACCCTTGAAAATGATGT (reverse primer). Sequences used for human GAPDH were CAAGCTTCCCGTTCTCAGCC (probe), ACCTCAACTACATGGTTTAC (forward primer) and GAAGATGGTGATGGGATTTC (reverse primer). A generalized linear mixed model with the log link function, a fixed group effect (CQ vs. CTL) and a random experiment effect (experiment date) was fit to the data (*n* = 3 in triplicate), allowing for group-specific over-dispersion. Statistical analysis was conducted using the SAS software (version 9.3, Cary, NC, USA).

Real-time RT-PCR studies were conducted to determine the mRNA levels of OATP1B1 following chloroquine treatment. As shown in Figure 1, treatment with chloroquine does not significantly affect the mRNA levels of OATP1B1, suggesting that reduced transport function of OATP1B1 following chloroquine treatment is not likely to occur at the transcriptional level. 

### 8.2. Regulation of OATP1B1 and OATP1B3 Transport Function by Proteasome Inhibitors

One of the important roles of ubiquitin is to form poly-ubiquitin tags on a protein and mediate its recognition and subsequent degradation by the proteasome [164]. Due to the important role of the ubiquitin system in the regulation of diverse cellular processes and its relationship to disease, the proteasome has emerged as a new therapeutic target [165,166,167,168,169,170]. Bortezomib is the first-in-class proteasome inhibitor approved as the first-line treatment option for multiple myeloma and second-line treatment option for mantle cell lymphoma [171]. Although OATP1B1 and OATP1B3 are ubiquitinated proteins in transporter overexpressing HEK293 cells [138], treatment with bortezomib did not affect the total protein levels of OATP1B1 and OATP1B3, suggesting that the proteasome is likely to play a minor role in degradation of OATP1B1 and OATP1B3 under constitutive conditions. 

Bortezomib is not a competitive inhibitor of OATP1B1 or OATP1B3 at clinically relevant concentrations up to 98 nM [138]. Interestingly, pretreatment with bortezomib decreased OATP1B3-mediated transport in a substrate-dependent manner. Treatment with bortezomib decreased transport of CCK-8, a specific substrate of OATP1B3 in HEK293 cells overexpressing OATP1B3 and in human SCH. Bortezomib treatment did not affect transport of pitavastatin and/or estradiol 17β-d-glucuronide mediated by OATP1B1 or OATP1B3. Pretreatment with other proteasome inhibitors MG132, epoxomicin and carfilzomib also significantly decreased OATP1B3-mediated transport of CCK-8. 

A pharmacoepidemiological study was conducted using myopathy data from the FDA Adverse Events Reporting System (FAERS) to test whether bortezomib plus metabolically stable statins (pitavastatin, rosuvastatin and pravastatin) or all statins (pitavastatin, rosuvastatin, pravastatin, simvastatin, atorvastatin, fluvastatin and lovastatin) leads to higher myopathy risk than do these statins alone [161,172,173]. Two groups of statins were used in this study (Table 2). One group of statins contained all seven statins currently on the market (simvastatin, lovastatin, fluvastatin, atorvastatin, pitavastatin, rosuvastatin and pravastatin); the other group contained only the three metabolically stable statins currently on the market (pitavastatin, rosuvastatin and pravastatin) [59,174,175]. As shown in Table 2, metabolically stable statins and all statins alone led to myopathy risks of 9.2% and 8.8%, respectively, without concurrent administration of bortezomib, while co-administration of bortezomib with all statins and metabolically stable statins led to myopathy risks of 8.0% and 8.6%, respectively. There were no significant differences in myopathy risk in patients concurrently using bortezomib and statins and patients using only statins (Table 2). Both published in vitro findings [138] and current pharmacoepidemiologic studies suggest that bortezomib is unlikely to cause OATP-mediated DDIs against statins. 

## 9. Discussion and Conclusions

**Figure 2:**
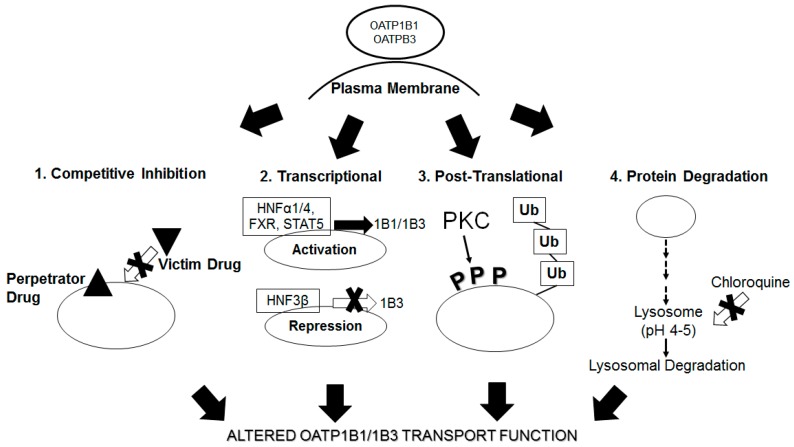
Summary of regulation of OATP1B1 and 1B3 transport function. OATP1B1 and 1B3 transport proteins can be regulated at the (**1**) plasma membrane by competitive inhibition; (**2**) by transcriptional factors than can repress or activate transcription; (**3**) post-translational modification by phosphorylation (P) or ubiquitination (Ub) and (**4**) by alteration of protein degradation. All four mechanisms are important for the regulation of OATP1B1 and OATP1B3 optimal transport function of its substrates.

Currently, assessing OATP-mediated DDIs in vitro using a static or dynamic model prediction is largely based on the assumption of competitive transporter inhibition by perpetrator drugs [8]. Increasing evidence suggests that transport functions of OATP1B1 and OATP1B3 can be altered by therapeutic drugs at levels other than competitive inhibition. Figure 2 summarizes what was described in this article regarding how drugs/chemicals may alter OATP1B1 and OATP1B3 transport function via altered gene transcription, phosphorylation, ubiquitination and altered protein degradation. In addition to what is summarized in this review, recent reports indicate that pretreatment with some OATP inhibitors such as CsA, rifampicin and dasatinib reduces OATP1B1- and OATP1B3-mediated transport even after washing out these inhibitors from incubation buffer [78,176,177,178]. Following pretreatment, the in vitro inhibition constant (*K*_i_) values for these inhibitors against OATP1B1 and OATP1B3 were reduced [78,176,177]. For CsA A and rifampicin, the in vitro *K*_i_ values determined after pretreatment were close to the estimated in vivo *K*_i_ values [78,176,177]. The pre-incubation step with inhibitors has been incorporated into the most recently published US FDA draft guidance for assessing in vitro OATP1B1- and OATP1B3-mediated DDI studies [10]. A recent review article has summarized the pre-incubation effects [179] on OATP1B1- and OATP1B3-mediated transport. Several potential mechanisms have been proposed to explain the underlying pretreatment inhibitory effects on OATP1B1 and 1B3, including post-translational regulation of the transporter proteins. 

The current knowledge gap regarding post-translational regulation on OATP1B1 and OATP1B3 function includes the lack of information on the functional phosphorylation and ubiquitination sites of the transporters. Methodologies of using mass spectrometry to characterize the phosphorylation/ubiquitination sites followed by site-specific mutagenesis has been utilized to study organic anion transporters (OAT) post-translational regulation [180]. A similar method can be applied to characterize the functional phosphorylation and/or ubiquitination sites of OATP1B1 and OATP1B3 proteins that are important for their regulation. In addition, studying the association of drug pretreatment with altered post-translational modification of OATP1B1 and OATP1B3 may shed light on if post-translational regulation may play a role in such pretreatment induced inhibitory effects. Knowledge gained from these studies will ultimately help to elucidate mechanism(s) underlying the OATP-related DDIs and serve as a foundation for predicting potential OATP-related DDIs. 

## Figures and Tables

**Table 1:** Summary of observed AUC ratio (AUCR) of clinical substrates of OATP1B1/1B3.

Substrate	Perpetrator Drugs	OATP1B1 Inhibition	OATP1B3 Inhibition	Reported AUCR
Atorvastatin	Boceprevir	[64]	[64]	2.3 [65]
	Faldaprevir	[66]	[66]	9 [67]
	Sacubitril/Valsartan	[68,69]	[68,69]	1.3 [70]
	Simprevir	[71]	[71]	2.1 [72]
	Telaprevir	[73]	[73]	7.8 [74]
	Tipranavir/Ritonavir	[75]	[76]	9.4 [77]
Pitavastatin	Rifampicin	[78]	[78]	5.7–7.6 [79], 5.7 [80]
Pravastatin	Boceprevir	[64]	[64]	1.6 [65]
	Daclatasvir/Beclabuvir/Asunaprevir cocktail	[81]	[81]	1.7 [82]
	Darunavir/Ritonavir	[75,83]	[76,83]	2.1 [84]
	Paritaprevir/Ritonavir/Ombitasvir/Dasabuvir	[85]	[85]	1.8 [86]
Repaglinide	Clopidogrel	NA	[87]	3.1 [88]
	Gemfibrozil	[89]	[90]	1.8 [91], 3.4 [92]
	Rifampicin	[78]	[78]	1.9 [93]
Rosuvastatin	Elvitegravir/Cobicistat/Emtricitabine/Tenovfovir	[94]	[94]	1.4 [94]
	Faldaprevir	[66]	[66]	15 [67]
	Fostamatinib	[95]	NA	2.0 [96]
	Furosemide/Digoxin/Metformin	[97]	[22,97]	1.4 [98]
	Paritaprevir/Ritonavir/Ombitasvir/Dasabuvir	[85]	[85]	1.6 [86]
	Rifampicin	[78]	[78]	2 [99], 4.6–5.2 [79], 5 [100]
	Simprevir	[71]	[71]	2.8 [72]
	Telmisartan	[101]	[101]	1.3 [102]

NA: not reported.

**Table 2:** DDI-myopathy analysis.

Drugs	Number of Patients Taking Drug (*N*)	Number of Myopathy (*M*)	Risk	Relative Risk/*p*-Value
Metabolically stable statins ^†^	88,682	8149	9.2%	
Bortezomib and metabolically stable statins ^†^	311	25	8.0%	0.87/0.58
All statins ^§^	339,094	29,910	8.8%	
Bortezomib and all statins ^§^	1296	112	8.6%	0.98/0.87

^†^ Statins: rosuvastatin, pravastatin and pitavastatin; ^§^ Statins: atorvastatin, simvastatin, lovastatin, fluvastatin, rosuvastatin, pravastatin and pitavastatin. *N* and *M* represent sum of counts for each individual statin. Risk is calculated as *M*/*N* × 100%. Relative risk is calculated as risk (bortezomib and statins)/risk (statins alone). Statistical analysis was performed with Chi-square test. All of the adverse event case reports from quarter 1 of 2004 to quarter 3 of 2012 were used for data analysis (*n* = 6.47 million).
